# Integration of synthetic aperture radar and optical satellite data for corn biomass estimation

**DOI:** 10.1016/j.mex.2020.100857

**Published:** 2020-03-13

**Authors:** Mehdi Hosseini, Heather McNairn, Scott Mitchell, Laura Dingle Robertson, Andrew Davidson, Saeid Homayouni

**Affiliations:** a Geomatics and Landscape Ecology Laboratory, Department of Geography and Environmental Studies, Carleton University, Ottawa, Canada; b Ottawa Research and Development Centre, Agriculture and Agri-Food Canada, Ottawa, Canada; c Department of Geographical Sciences, University of Maryland, College Park, MD, USA; d Centre Eau Terre Environnement, INRS, Quebec, Canada

**Keywords:** SAR, Optical, Integration, Biomass, Neural network

## Abstract

Efforts to use satellites to monitor the condition and productivity of crops, although extensive, can be challenging to operationalize at field scales in part due to low frequency revisit of higher resolution space-based sensors, in the context of an actively growing crop canopy. The presence of clouds and cloud shadows further impedes the exploitation of high resolution optical sensors for operational monitoring of crop development. The objective of this research was to present an option to facilitate greater temporal observing opportunities to monitor the accumulation of corn biomass, by integrating biomass products from Synthetic Aperture Radar (SAR) and optical satellite sensors. To accomplish this integration, a transfer function was developed using a Neural Network algorithm to relate estimated corn biomass from SAR to that estimated from optical data. With this approach, end users can exploit biomass products to monitor corn development, regardless of the source of satellite data.

• The Water Cloud Model (WCM) was calibrated or parametrized for horizontal transmit and horizontal received (HH) and horizontal transmit and vertical received (HV) C-band SAR backscatter using a least square algorithm.
• Biomass and volumetric soil moisture were estimated from dual-polarized RADARSAT-2 images without any ancillary input data.
• A feed forward backpropagation Neural Network algorithm was trained as a transfer function between the biomass estimates from RADARSAT-2 and the biomass estimates from RapidEye.

## Graphical abstract

**Figure:**
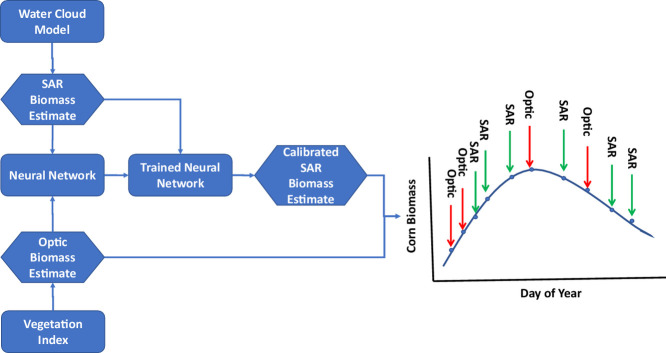

**Table:** Specification table

Subject Area	*Agricultural and Biological Sciences*
More specific subject area:	*C*rop biophysical parameters modeling
Method name:	*Empirical model, semi-empirical model, machine learning model*
Name and reference of original method	*Water Cloud Model* [1]*. Vegetation modelled as a water cloud. Radio Science, Vol. 13, pp. 357–364.*
Resource availability	https://smapvex12.espaceweb.usherbrooke.ca/intranet.php

## Method details

The Water Cloud Model (WCM) is a semi-empirical model that has been frequently used by researchers to estimate crop biophysical parameters from SAR data [2,5,8]. The compact form of the model is introduced in Eq. (1) [4].

(1) $$\sigma^{0}=AL^{E_{1}}\cos\theta \left(1-\exp\left(-\frac{2BL^{E_{2}}}{\cos\theta }\right)\right)+\left(CM_{v}+D\right)\;\times \;\text{exp}\left(-2BL^{E_{2}}/\cos\theta \right)$$

where *σ*^0^ is total backscatter in power unit, *L* is biomass, *M_v_* is volumetric soil moisture, *θ* is the incidence angle, and *A, B, C, D, E*_1_ and *E*_2_ are the coefficients.

The WCM model has six coefficients (*A, B, C, D, E*_1_ and *E*_2_) and two unknown variables (i.e. biomass and volumetric soil moisture). The model calibration to parameterize the six coefficients and its inversion to estimate the biomass and soil moisture are explained in the following sections.

### WCM model calibration

The WCM model has six coefficients and therefore, calibration of the model requires at least six calibration points with their ground measurements (i.e. biomass and soil moisture) and satellite observations (i.e. backscatter and incidence angle). However, to develop a robust model more data are needed over a wide range of biomass and soil moisture conditions. In this research, 23 calibration points were used with soil moisture ranging from 0.039 $m^{3}\;m^{-3}$ to 0.379 $m^{3}\;m^{-3}$, dry biomass from 0.003 $\text{kg}\;m^{-2}$ to 1.16 $\text{kg}\;m^{-2}$, wet biomass from 0.04 $\text{kg}\;m^{-2}$ to 7.1 $\text{kg}\;m^{-2}$ and SAR incidence angles from 21.025° to 31.9592°. A least square method [7] was used to calibrate the WCM model. To run the least square method, the *nlinfit* function in MATLAB (version R2018b) was used to estimate the six coefficients.

[Beta,R]=nlinfit(X,Y,@ModelFun,Beta0)

In the above code, *nlinfit* is a function that applies the least square method to a non-linear regression function and estimates its coefficients. Beta is the vector of estimated coefficients and its size is 6 × 1 in this study. R is the vector of residuals (6 × 1) for the estimated coefficients. X is the matrix of independent variables including biomass, soil moisture and incidence angle. The size of this matrix is 23 × 3. Y is a vector (23 × 1) of the dependent variable, in this study, total backscatter. ModelFun is the function for the WCM model. Beta0 is the vector (6 × 1) of initial values for the six coefficients. In this study, the initial values of the coefficients were random numbers between 0 and 1. The *nlinfit* function works based on an iterative approach, improving the initial coefficients (i.e. Beta0) in every iteration. The iteration terminates when the sum of squares of the residuals reaches its default tolerance value of 10^−8^, or the number of iterations reaches 100.

### WCM model inversion

A goal of this research was to estimate biomass and soil moisture by inverting the WCM model without the requirement of any additional input data. Because the WCM model has two unknown variables (i.e. biomass and soil moisture), the model was calibrated or parameterized for two polarizations - HH and HV. With these two equations (i.e. one for each of the polarizations), both biomass and soil moisture can be simultaneously derived using the Levenberg-Marquardt algorithm [6]. Using the *fsolve* function in MATLAB, this algorithm was implemented for all calibration and validation points.

V=fsolve(@Fun,V0)

V is the estimated variables (i.e. biomass and soil moisture) and is a vector of 2 × 1. V0 is the initial values for the estimated variables and has the same dimensions as V. In this study, the initial values for biomass and soil moisture were 1 $\text{kg}\;m^{-2}$ and 0.2 $m^{3}\;m^{-3}$, respectively. Fun is a system of two WCM equations (one for each polarization). The *fsolve* function, like the *nlinfit* function, needs initial values for the variables, improving these initial values with every iteration. The iterations stopped when the difference between the derived variables of the two iterations is less than 10^−6^, or the number of iterations reached 400.

### Calibration of optical models

The optical models (Table 1) were based on four vegetation indices - Normalized Difference Vegetation Index (NDVI), Red-Edge Triangular Vegetation Index (RTVI), Simple Ratio (SR) and Red-edge Simple Ratio (SRre). These indices were applied to reflectance data from RapidEye imagery. As with the calibration of the WCM model, the *nlinfit* function in MATLAB was used to calibrate the optical models. In this function, X is a 23 × 1 vector of the vegetation index and Y is a 23 × 1 vector of biomass measurements. ModelFun is the optical model (Table 1). Beta0 is the vector of initial values for the two coefficients and its size is 2 × 1. The initial values of the coefficients were random numbers between 0 and 1. As before, estimation of the coefficients was done iteratively. The iteration stopped when the sum of squares of the residuals reached to the tolerance value of 10^−8^, or the number of iterations reached 100.

**Table 1:** Biomass models based on optical vegetation indices. *a*_1_, *a*_2_, *a*_3_, *a*_4_, *b*_1_, *b*_2_, *b*_3_ and *b*_4_ are empirically derived coefficients. Separate sets of coefficients were estimated for wet and dry biomass.

Optical Models	
Normalized Difference Vegetation Index (NDVI)	$Biomass=a_{1}\times \exp\left(NDVI\right)+b_{1}$
Red-Edge Triangular Vegetation Index (RTVI)	$Biomass=a_{2}\times RTVI+b_{2}$
Simple Ratio (SR)	$Biomass=a_{3}\times \ln SR+b_{3}$
Red-edge simple ratio	$Biomass=a_{4}\times SRre+b_{4}$

### Transfer function

A transfer function between the biomass estimates from RADARSAT-2 and the biomass estimates from RapidEye was developed. The purpose of this function is to allow users to derive biomass from satellite data regardless of the source. The transfer function was a two-layer feed-forward backpropagation Neural Network model with 10 hidden neurons [3]. To train the model, the biomass estimates from RADARSAT-2 (from the calibration points) were used as input with the corresponding estimates from RapidEye as output. The model was trained with Levenberg-Marquardt algorithm using the MATLAB Neural Net Fitting tool. 70% of the calibration points (i.e. 17 points) was used to develop the Neural Network, with the remainder (6 points) reserved to validate the trained model. After the network was developed, it was used to adjust the SAR-based biomass estimates for the 43 validation points, using the following MATLAB code:

K=abs(NNModel(L))

K is the biomass estimates from the Neural Network model (a vector of 43 × 1). NNModel is the trained Neural Network function. L is the input to the Neural Network which contains the biomass estimates from the WCM model. The *abs* function delivers the absolute value of the estimate.

## Supplementary material

The measured dry and wet biomass, measured soil moisture, satellite observations including HH and HV backscatters and incidence angles and NDVI are reported for all the 66 points (including calibration and validation points) in Table 2. This table was sorted such that the first 23 points are the calibration points and the rest of the points were used as the validation points.

**Table 2:** Ground measurements and satellite observations for all the calibration and validation points.

Point No.	Dry biomass ($kg\;m^{-2}$)	Wet biomass ($kg\;m^{-2}$)	Soil moisture ($m^{3}\;m^{-3}$)	HH backscatters	HV backscatters	Incidence angles (degree)	NDVI
1	0.00799	0.08754	0.127239	0.620443	0.008223	27.1885	0.309056
2	0.00393	0.04304	0.267865	0.316343	0.028498	27.1032	0.276754
3	0.0149	0.16314	0.242614	0.144861	0.014744	27.0845	0.336444
4	0.00515	0.05644	0.139916	0.183946	0.006655	27.02	0.304782
5	0.00434	0.04754	0.171731	0.229378	0.006805	27.0479	0.194855
6	0.01732	0.18974	0.274307	0.407447	0.021909	27.0271	0.413577
7	0.0111	0.12154	0.170147	0.383534	0.008734	27.0894	0.334782
8	0.03721	0.40754	0.343364	0.320011	0.020286	26.8304	0.474459
9	0.06584	0.7211	0.107088	0.110205	0.008403	21.1371	0.222073
10	0.09773	0.6371	0.07947	0.086696	0.006914	21.0813	0.254141
11	0.12586	0.9439	0.039161	0.177344	0.007373	21.1235	0.516357
12	0.22256	1.6691	0.111372	0.085646	0.004978	21.2044	0.521337
13	0.24906	1.8679	0.224185	0.091497	0.006946	21.1843	0.642726
14	0.23056	1.7291	0.075383	0.148071	0.01253	21.1276	0.720683
15	0.19909	1.4931	0.110798	0.153117	0.009832	21.025	0.656
16	0.16399	1.2299	0.113555	0.082712	0.009516	21.0829	0.591136
17	0.41859	2.7287	0.075	0.144633	0.013843	31.9592	0.855379
18	0.60758	3.9607	0.119	0.159121	0.013454	31.8798	0.865662
19	0.84229	5.4907	0.076	0.160248	0.011591	31.8639	0.94174
20	0.71079	4.2917	0.107	0.208396	0.017317	31.8703	0.958239
21	1.0908	7.1107	0.082	0.15254	0.008089	31.6379	0.97088
22	0.53886	3.5127	0.1433	0.142494	0.014639	31.6591	0.978951
23	1.15769	6.1246	0.379	0.132422	0.01522	31.8169	0.984188
24	0.01205	0.13194	0.753	0.598379	0.014348	27.1802	0.327934
25	0.00718	0.07864	0.858	0.56054	0.010941	27.1769	0.32417
26	0.01246	0.13644	0.4208	0.184188	0.014659	27.0878	0.339747
27	0.00839	0.09194	0.1828	0.2243	0.016958	27.0764	0.250504
28	0.00962	0.10534	0.1617	0.230843	0.01999	27.0732	0.288926
29	0.00394	0.04314	0.1166	0.154635	0.004993	27.0291	0.276767
30	0.0019	0.02084	0.1401	0.131433	0.004617	27.0312	0.266659
31	0.00678	0.07424	0.1701	0.170975	0.006162	27.03	0.22734
32	0.00394	0.04314	0.1689	0.184585	0.006525	27.0336	0.152364
33	0.00555	0.06084	0.4197	0.32861	0.026821	27.0371	0.477624
34	0.01286	0.14084	0.1934	0.453664	0.013668	27.0399	0.374354
35	0.02224	0.24354	0.3655	0.370459	0.014349	26.8139	0.467909
36	0.01584	0.17354	0.2982	0.289838	0.01087	26.8169	0.381634
37	0.01968	0.21554	0.2814	0.141431	0.015722	26.8713	0.395569
38	0.02443	0.26754	0.3684	0.149793	0.020624	26.8563	0.429161
39	0.087419	0.9575	0.638	0.104647	0.006665	21.1178	0.376021
40	0.0962	0.7215	0.1007	0.105227	0.005444	21.1225	0.436973
41	0.14903	0.9715	0.0606	0.120936	0.007743	21.0913	0.084151
42	0.121004	0.9075	0.0846	0.108791	0.007812	21.0942	0.115846
43	0.19327	1.4495	0.0585	0.167893	0.011137	21.1418	0.637158
44	0.23114	1.7335	0.0419	0.152627	0.01108	21.1376	0.544374
45	0.251408	1.8855	0.1633	0.072711	0.004638	21.2143	0.493171
46	0.136738	1.0255	0.202	0.105992	0.008347	21.2181	0.58312
47	0.190606	1.4295	0.0815	0.07359	0.00436	21.203	0.453194
48	0.139138	1.0435	0.0738	0.081103	0.005024	21.1989	0.514886
49	0.109537	0.8215	0.1927	0.107881	0.008487	21.0044	0.770156
50	0.22847	1.7135	0.042	0.143742	0.008616	21.0082	0.576924
51	0.49835	3.7375	0.0559	0.144683	0.016928	21.1112	0.805761
52	0.18927	1.4195	0.0382	0.13543	0.01355	21.115	0.698382
53	0.34341	2.5755	0.0833	0.096725	0.011773	21.0582	0.699992
54	0.31461	2.3595	0.0581	0.105631	0.013265	21.0743	0.719998
55	0.70627	4.604	0.0814	0.192856	0.01323	31.9515	0.95791
56	0.67497	4.4	0.1109	0.198	0.013736	31.9487	0.925892
57	0.526479	3.432	0.0985	0.121614	0.011253	31.8637	0.905323
58	0.75658	4.932	0.0514	0.162414	0.01141	31.8661	0.911178
59	0.60195	3.924	0.0707	0.127441	0.009791	31.8563	0.87577
60	0.69123	4.506	0.0661	0.120812	0.01094	31.8536	0.900459
61	0.95631	6.234	0.0589	0.146173	0.012034	31.6233	0.980786
62	0.48352	3.152	0.1074	0.164818	0.012403	31.626	0.959513
63	0.589067	3.84	0.1173	0.151315	0.011064	31.6761	0.972097
64	0.534456	3.484	0.1144	0.161151	0.017059	31.6627	0.974763
65	0.6214	3.752	0.4348	0.165078	0.014831	31.826	0.963522
66	1.166288	7.042	0.4163	0.152524	0.013868	31.8287	0.972115

## Declaration of Competing Interest

The authors declare that they have no known competing financial interests or personal relationships that could have appeared to influence the work reported in this paper.
